# Study Habits in Medical Education: Examining How German Medical Students Study Using a Cross-sectional Mixed-Methods Survey

**DOI:** 10.1007/s40670-025-02324-9

**Published:** 2025-02-15

**Authors:** Sophia Viktoria Ragaller, Johanna Flora Rother, Alexandra Aster, Tobias Raupach

**Affiliations:** Institute of Medical Education, University Hospital Bonn, Venusberg-Campus 1, 53127 Bonn, Germany

**Keywords:** Study behaviour, Assessment-oriented learning, Achievement goal theory, Learning strategies, Medical education

## Abstract

The shift to digital teaching and learning during the pandemic has had a lasting impact on how students study medicine. Understanding how medical students learn and which resources they use is essential for successfully implementing teaching innovations. In this cross-sectional survey at a German medical school, 340 students completed the LIST-K questionnaire to assess cognitive, metacognitive, and resource management learning strategies. Additionally, students provided information on their preferences regarding educational media and tools used during their study process. Data were analysed with a specific focus on differences between preclinical and clinical years of medical education. Students primarily used resource management strategies, such as effort and external sources of information, while cognitive strategies such as organisation and critical review were employed less frequently. There was a strong emphasis on using local exam questions and a commercial product providing past exam questions and supplemental content. When examining media and tool use in different phases of medical education, the most significant difference related to a less frequent use of textbooks and other literature in clinical compared to preclinical years. Our results highlight students’ focus on assessment-oriented learning, particularly the use of past exam questions, suggesting a performance goal orientation rather than a mastery approach. However, due to its cross-sectional design, the study does not allow causal conclusions to be drawn. The heavy reliance on a commercial product not aligning with the local curriculum is concerning, and future studies should explore underlying reasons and potential interventions to address this unbalance.

**Supplementary Information:**

The online version contains supplementary material available at 10.1007/s40670-025-02324-9.

## Introduction

In a challenging curriculum such as medicine, it is important that educators and institutions support the students’ motivation and help them study to their best abilities. The shift into a more digital education following the covid-19 pandemic, which has significantly influenced not only educational practices, but also different aspects of student performance and engagement [1], has led to a rising number of digital study tools. This begs the question whether real-life students actually prefer these digital tools over traditional methods when it comes to studying and what they use to achieve their academic goals.

A theory that expands on the thought of individuals engaging in certain behaviours for different reasons is the *achievement goal theory* [2], according to which goals can be classified into two categories. While a person following a performance goal desires to demonstrate ability to others to seem competent, mastery goals relate to mastering a task and acquiring skills without having the need to prove the competence to other people and without outperforming others [3, 4]. There have been findings which associate mastery goals with enhanced intrinsic motivation [5], which describes the thought of wanting to reach a goal without external pressure or the need to demonstrate competence to others. Findings regarding the impact of goal settings are rather heterogeneous and suggest that different environments or participants can benefit from different goal settings when it comes to their academic achievements [6, 7].

For students, there has been research about whether learning orientations and strategies can be classified using a similar terminology and what influences them. The summarising term *assessment drives learning* [8] describes the concept that students’ learning behaviours are mostly driven by their exams and assessments, which highlights the importance of adequate examination. Another term is *examination-oriented learning* where the main focus is on (mostly) end of period exams to evaluate the students and their achievement, without considering personal factors such as a students’ transformation or condition [9]. It has also been hypothesised that, in this context, an unintended effect of exams could be that students prefer superficial knowledge in order to cram for exams, without reflecting the materials [9, 10]. Surface learning is characterised by studying and learning without gaining a true understanding of the content and only aiming to reproduce learned material in an exam situation [11, 12], while deep learning is related to intrinsic interest and aims at a deeper understanding of the subject matter [12, 13]. Previous studies specifically targeting medical students and practising physicians [14] have identified and validated not only these two learning strategies, but a third study type: the strategic approach. When students apply this approach, they strategically modify their study habits to achieve academic success [11, 15]. A cross-sectional study, which examined learning strategies in Irish medical students, found differences in study strategies between undergraduate and graduate entrants, with graduate students being less likely to take a surface approach [11, 16]. Regarding the outcomes, the surface strategy has been associated with poorer academic performance [11], while the deep and strategic approaches have been found to have an advantage over the surface approach in relation to academic achievement [17]. Therefore, it might be important to discourage surface learning and promote deep and strategic learning to improve the students’ achievements [17]. Unfortunately, the multiple-choice approach which is popular in many study courses, including medicine, has been shown to rather encourage surface learning, while deep learning strategies even correlated with a poorer performance in multiple-choice examinations [18].

Another popular classification regarding learning strategies was developed by Wild and Schiefele [19]. It differentiates between cognitive, metacognitive and resource management strategies and aims at categorising different learning mechanisms, such as rehearsal, planning, and attention management into the three broader categories. While rehearsal falls into the category of cognitive strategies, since it deals with the processing and storage of information, the metacognitive strategies, such as planning, are meant to control and monitor learning and study sessions. Lastly, resource management strategies are meant to provide internal and external factors which help provide adequate resources to the learner, for example through attention management (internal) or a positive study environment (external) [19].

Recent research mainly drew on the framework of deep, surface, and strategic approach [11, 16, 17]. An exception is the longitudinal study by Hogh and Müller-Hilke [20] which employed the LIST questionnaire (Learning Strategies of University Students; [19]) to assess the strategies in German medical students during their first and second preclinical years. More details on the questionnaire can be found in the “Method” section. Hogh and Müller-Hike identified four different patterns of strategy combinations (the relaxed learners, the diligent learners, the hard-working learners, and the sociable learners) and investigated the changes during the year. The patterns were defined by different combinations of learning strategies, depending on the different scales’ manifestations. Nonetheless, this study examined a relatively small sample of *N* = 53 and did not examine the learning activities individually. Additionally, they did not examine students in the clinical years, which begs the question whether the strategies differ between preclinical and clinical years. A study which laid a focus on the differences between preclinical and clinical years was conducted by Cordovani et al. [21]. They examined motivation and learning strategies of Canadian medical students using the Motivated Strategies for Learning Questionnaire (MSLQ) which includes scales related to cognitive, metacognitive, and resource management strategies in addition to motivational aspects. In terms of learning strategies, the highest scores were observed for the scale elaboration, while rehearsal (memorisation) appeared to be the least utilised strategy for this sample. A comparison of MSQL scores by academic year revealed significantly lower scores for the scale effort regulation among first-year students compared to third-year students [21]. The results of this study suggest that different learning approaches may be employed during the preclinical and clinical years, highlighting the need for further research with larger sample sizes and different educational systems.

In relation to the summarised findings, it seems essential to assess not only the learning strategies and general orientations, but also the specific resources the students use. Previous studies conducted in countries such as Australia [22] revealed that medical students preferred the traditional resources such as attendance at lectures for learning new information, but also frequently used medical apps and online question apps. They also reported that the students preferred different resources, depending on whether they were seeking to learn new content or revise previously learned content [22].

Summarising these findings, there is a research gap regarding learning strategies and use of learning tools in German medical students across the entire curriculum. In order to adequately assess these factors, it also seems essential to factor in the current year that the students are in.

Therefore, our research questions (RQ) are:

1. Which learning strategies are primarily used by medical students?
2. Which media and tools are predominantly used by medical students?
3. Are there any differences between students in the preclinical years (1st to 4th term) and students in the clinical years (5th to 10th term) regarding their study behaviour (learning strategies and tools used)?

## Method

### Sample

Study participants were medical students at Bonn Medical School. Its undergraduate curriculum is divided into three main study sections: two preclinical years, three clinical years, and the practical year. For this study, students in their first two study sections were included. The preclinical years focus on acquiring basic knowledge in fields such as biology and human anatomy, biochemistry, and physiology. In the clinical years, the emphasis is on in-depth, practical training in clinical diagnosis and treatment. Both the preclinical and clinical years conclude with a nationwide Medical State Examination developed and coordinated by the German National Institute for State Examinations in Medicine, Pharmacy and Psychotherapy (IMPP), referred to as the first (M1) and second high stakes exam (M2) in the following.

### Survey Tool

The data collection took place during summer term 2024 via an online survey hosted on EvaSys (Electric Paper, Lüneburg, Germany), using a mixed-methods questionnaire with both multiple-choice and open questions (see Table 2 in Appendix). Students received the link to the study via mail. In addition to that, the study coordinate presented the content and aims of the study and a corresponding QR code in some lectures. The survey included sociodemographic and study-related questions (gender, current year of study, preparation for high stake exams), students’ learning strategies, and items asking about media and tools used during the study process for each of three phases (during term, exam preparation, state exam preparation).

To measure the students’ learning strategies, the short version of the questionnaire *Learning Strategies of University Students* (LIST-K; [19, 23]) was used. It consists of 39 items regarding cognitive, metacognitive, and (internal and external) resource management strategies, which are rated on a 5-point scale from 1 (very rare) to 5 (very often). In total, there are 13 scales referring to one learning strategy each which will be used as proposed by Klingsieck [23]. Cognitive strategies include *elaboration*, *organisation*, *critical review*, and *repetition* (memorisation) of learning content. Metacognitive strategies encompass *regulation* (i.e. adapting study behaviour in response to challenges), *control* (i.e. quiz oneself on the study material), and *goals and planning*. Internal resource management strategies focus on managing internal resources like *attention*, *effort*, or *time*. In contrast, external resource management strategies aim at external factors like choosing a productive *learning environment*, *learning with fellow students*, or consulting *external sources of information*. The last scale was changed from the original wording *literature research* to the more general term *external sources of information*, since the items on this scale are not clearly specified to only include (scientific) literature. Exemplarily, in item Lit2 (“I gather missing information from different sources [e.g. lecture notes, books, scientific journals]”), looking up information on the Internet could be considered as a possible source as well.

For the items regarding most used media and tools, the answer options include some commonly used resources such as lecture and seminar material, previous local exam questions, conventional and digital flashcards, textbooks, and other literature as well as commercial products (CP) providing past exam questions and supplemental content like Amboss (CP1) or ViaMedici (CP2). For the CP1, participants can choose between the two separate answer options regarding the past exam questions on the one hand and the supplemental content on the other hand. The reason for this differentiation was to consider the different natures of using the digital tool to look up content versus testing oneself with former high stake exam questions.

### Statistical Analyses

Descriptive analyses (e.g. arithmetic mean, standard deviation) were used to answer RQ1 and RQ2. To answer RQ3, mean differences between learning strategies of students in the preclinical years vs. students in the clinical years were examined by means of t-tests. In case of a violation against the assumption of normal distribution, Mann–Whitney-U-tests were used instead. Effect sizes are reported as Cohen’s d. Additionally, Chi-square-tests were used to examine the differences between students in the preclinical vs. clinical years regarding the media and tools used (separately for every tool and the two study phases during term vs. during exam preparation). Effect sizes are reported as Cramer’s V. A power calculation using the program G*Power revealed that for the *t*-tests, there should be at least 105 participants in each group. Therefore, our goal was to reach a minimum of 210 participants.

## Results

### Sample Characteristics

A total of *N* = 340 medical students participated in the survey (29% male, 69% female, 1% non-binary, 1% with missing data on gender). Just over half of the respondents stated to be in the preclinical years of their education (55%), while 43% were in the clinical years and 2% did not state their study phase. Of those who stated the term they were studying in, the majority of participants were enrolled in the second term (40%), followed by the seventh term (18%) and fourth term (15%); 28% were distributed over the remaining terms. Regarding the current preparation for high stake exams, 10% of those surveyed were preparing for the first and 6% for the second high stake exams. However, the vast majority of participants (84%) was not preparing for one of the high stake exams at the date of survey.

### RQ 1: Medical Students’ Learning Strategies

Overall, students mostly reported using the internal resource management strategy *effort* together with the external resource management strategies *external sources of information* and *learning environment*. The least reported strategies were the internal resource management strategy *time*, as well as the cognitive strategies *organisation* and *critical review*. When examining the individual study parts, a similar pattern emerges in the most and least used strategies. Students in the preclinical years mostly reported applying the external resource management strategy *external sources of information* and least reported applying the internal resource management strategy *time*. Students in the clinical years mostly reported applying the internal resource management strategies *effort* and least reported applying the internal resource management strategy *time* (see Table 1). Information on any statistical significances between preclinical and clinical years is available in RQ3.

**Table 1:** Medical students’ learning strategies

Overarching strategies	Scales	Overall (mean ± SD (*N*))	Preclinical years (mean ± SD (*N*))	Clinical years (mean ± SD (*N*))
Cognitive strategies	Repetition	3.42 ± 0.93 (327)	3.54 ± 0.91 (176)	3.34 ± 0.89 (144)
	Elaboration	3.29 ± 0.86 (337)	3.26 ± 0.88 (185)	3.31 ± 0.85 (145)
	Organisation	3.05 ± 1.09 (330)	3.11 ± 1.03 (178)	2.98 ± 1.17 (145)
	Critical review	2.98 ± 0.83 (335)	2.97 ± 0.84 (183)	3.00 ± 0.82 (145)
Metacognitive strategies	Regulation	3.67 ± 0.81 (335)	3.61 ± 0.81 (183)	3.72 ± 0.80 (145)
	Control	3.66 ± 0.77 (335)	3.74 ± 0.76 (183)	3.56 ± 0.78 (145)
	Goals and planning	3.49 ± 0.93 (335)	3.56 ± 0.91 (183)	3.37 ± 0.95 (145)
Internal resource management strategies	Effort	4.10 ± 0.67 (333)	4.07 ± 0.71 (181)	4.15 ± 0.62 (145)
	Attention	3.28 ± 0.95 (330)	3.28 ± 0.92 (178)	3.28 ± 0.98 (145)
	Time	2.73 ± 1.16 (335)	2.73 ± 1.13 (183)	2.72 ± 1.20 (145)
External resource management strategies	External sources of information	4.01 ± 0.86 (329)	4.11 ± 0.76 (177)	3.89 ± 0.96 (145)
	Learning environment	3.70 ± 0.78 (337)	3.70 ± 0.76 (185)	3.67 ± 0.80 (145)
	Learning with fellow students	3.11 ± 0.96 (335)	3.15 ± 0.98 (183)	3.09 ± 0.92 (145)

### RQ 2: Medical Students’ Most Used Media and Tools

Student responses on their use of media and tools are reported separately depending on when these resources were used: (a) *during term*, (b) *while preparing for local exams*, (c) *while revising for the high stake exams*. During term (i.e. when students first learned new material), the most used tool was lecture notes (*n* = 280, 82.6%), followed by the use of learning material provided by the CP1 (*n* = 261, 77%), questions from previous local exams (*n* = 222, 65.5%), and seminar material (*n* = 211, 62.2%). While preparing for local exams, students preferred using questions from previous exams (*n* = 296, 88.4%), followed by lecture material (*n* = 225, 67.2%) and learning material in the CP1 (*n* = 217, 64.8%).

For the preparation for high-stakes exams, students mostly used questions from previous high-stakes examinations provided in the CP1 (*n* = 39, 69.6%), followed by learning material also contained in the product (*n* = 37, 66.1%), digital flashcard apps (*n* = 24, 42.9%), and the CP2 (*n* = 20, 35.7%).

### RQ 3: Differences in Learning Behaviour During the Preclinical Versus Clinical Years

In a final set of analyses, we compared both learning strategies and media and tools used by students between preclinical and clinical students. Owing to a non-normal distribution of the data, Mann–Whitney *U*-tests were used to analyse the differences regarding learning strategies. The only significant difference referred to the scale *repetition* (*U* = 10,995.00, *Z* =  − 2.05, *p* = 0.04, *r* = 0.115) between preclinical (*M*_rank_ = 170.03) and clinical students (*M*_rank_ = 148.85). Descriptive statistics can be found in Table 1.

**Fig. 1:**
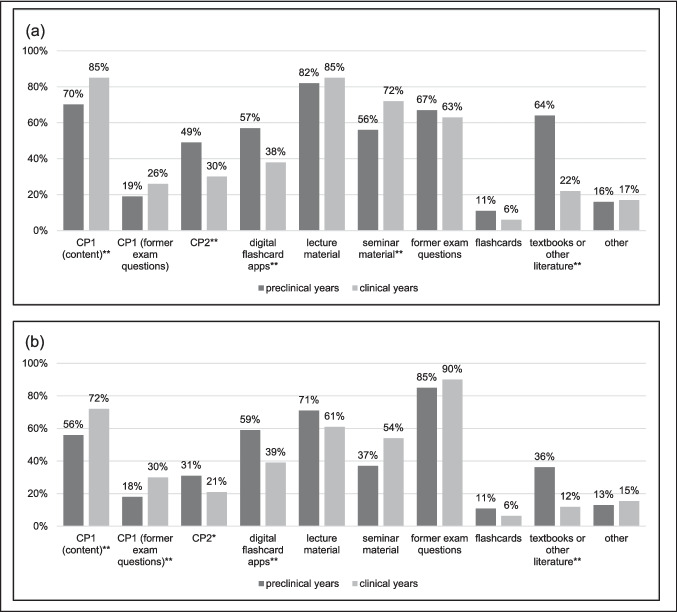
Most used media and tools preclinical vs. clinical years (multiple answers possible) **a** during term and **b** during preparing for local exams. *Note*. * < .05, ** < .01

With regard to the media and tools used by students during term, there were some significant differences between preclinical and clinical years. During term, learning material contained in the CP1 (*χ*^2^(1) = 8.77, *p* = 0.003, *V* = 0.16) and seminar material (*χ*^2^(1) = 8.83, *p* = 0.003, *V* = 0.16) were used more frequently in clinical years, while the use of the CP2 (*χ*^2^(1) = 11.71, *p* = 0.001, *V* =  − 0.19), digital flashcard apps (*χ*^2^(1) = 12.48, *p* < 0.001, *V* = 0.19), and textbooks (*χ*^2^(1) = 57.54, *p* < 0.001, *V* = 0.42) was significantly lower in clinical years. Regarding exam preparation, there was a similar trend with a significantly higher use of the CP1’s content (*χ*^2^(1) = 9.06, *p* = 0.003, *V* = 0.17) and previous high-stakes exam questions (*χ*^2^(1) = 6.81, *p* = 0.009, *V* = 0.14), seminar material (*χ*^2^(1) = 9.09, *p* = 0.003, *V* = 0.17), and a significantly lower use of the CP2 (*χ*^2^(1) = 4.78, *p* = 0.029, *V* =  − 0.12), digital flashcard apps (*χ*^2^(1) = 12.07, *p* = 0.001, *V* =  − 0.19), and textbooks (*χ*^2^(1) = 24.82, *p* < 0.001, *V* =  − 0.27) in clinical years. For both term and exam preparation, the effect was most pronounced for textbooks. Figure 1 depicts how the use of media and tools differs between preclinical and clinical students.

## Discussion

Our study investigated students’ learning behaviour and their most used tools. The results have to be interpreted in light of the recent covid-19 pandemic, which had a significant impact on educational practices around the world [1]. According to our data, study habits of German medical students are mainly characterised by resource management learning strategies like *effort* (internal resource) and *use of external sources of information* and *learning environment* (external resources) combined with the predominant use of certain tools (lecture material, previous local exam questions, and commercial digital tools). The least used learning strategies were found to be the internal resource management strategy *time* followed by the cognitive strategies *critical review* and *organisation*. Regarding the difference between preclinical and clinical years, the strongest effect was found for the use of textbooks or other literature which was much less prevalent in clinical years. At the same time, students in their clinical years showed a significantly lower use of the cognitive learning strategy *repetition*, i.e. learning content by heart, compared to students in their preclinical years.

A notable aspect of the most used strategies is the high prevalence of resource management strategies such as *external sources of information*. As mentioned earlier, the items belonging to this scale can be interpreted in a wider sense than solely researching literature. Hence, this finding does not contradict the fact that working with textbooks or other literature was not among the most used tools, especially in the clinical years. More precisely, it supports the idea that commercially available digital tools gain popularity and may even replace conventional tools such as textbooks. Our results suggest that students use material provided by the medical school (i.e. lecture and seminar handouts, previous exam questions) to the same extent as commercially available products that are not aligned to the local curriculum.

The lower mean on the scale *organisation*, i.e. a rarer use of this cognitive strategy that is related to the in-depth work with and preparation of learning material, indicates less willingness to contribute something themselves and instead preferring to use premade material (e.g. summaries). A possible reason for this could be a lack of time in a full schedule, but it might also hint at the fact that medical students focus on performance goals rather than mastery goals. In other words, students attribute less importance to acquiring specific expertise which could be gained while researching for and creating summaries and instead concentrate on studying the material to pass exams. This hypothesis of a higher prevalence of performance goals is also supported by the low means for the learning strategy *critical review*, because critically engaging with the study contents might be more important for mastery goals than for performance goals. Nonetheless, critical review is an important aspect in the study process, especially for developing a scientific understanding which is important for any practitioner. Ratte et al. [24] showed that German medical students evaluated scientific competencies like critically reviewing relevant literature as important for themselves. Since our study demonstrated rather lower mean values on the scale *critical review*, we assume a discrepancy between students’ self-perception and actual use of scientific competencies.

Furthermore, the results show a strong integration of previous local exam questions into the study process. Possible functions of using previous exam questions can be to check one’s level of knowledge, to identify knowledge gaps or to familiarise with the format of exam questions. However, applying this strategy comes with risks such as cueing effects (i.e. learning wrong answers as correct by reading them repeatedly) or limiting the study process on memorising answer options (as many questions are being used multiple times). Keeping the achievement goal theory in mind, the preferred use of previous exam questions could again implicate underlying performance goals. It also suggests assessment-oriented learning since the examination format is explicitly integrated into the study process. Testing oneself regularly can positively affect the retention of information, a phenomenon which is called the *testing effect* and increasingly examined in the context of medical education [25, 26]. In addition to the testing effect, other benefits of integrating frequent tests into medical education are discussed elsewhere (e.g. [27]).

The only significant mean difference in learning strategies between the preclinical and clinical years can be found for the learning strategy *repetition.* Items on this scale ask about memorising content, which is a classic example of a superficial learning strategy. The associated effect size is low, so the result has to be interpreted with care, but it suggests that students use this strategy less in the clinical years. A possible reason could be that the clinical years—by definition and conceptualisation—accentuates in-depth, practical training in clinical diagnosis and treatment while preclinical years emphasises the establishment of a strong (back-)ground knowledge. More practice-oriented forms of assessment such as objective structured clinical examinations (OSCE) are found in this study section which usually examine practical and clinically relevant skills such as doctor-patient communication or examination techniques [28]. This aligns with the concept of assessment-driven learning indicating that the implementation of more practice-oriented exams indeed affects the study behaviour of medical students in a sense that less superficial strategies are used. Nevertheless, since we only found a small effect size for the difference between the preclinical and clinical years, further research is necessary. In contrast to the study of Cordovani et al. [21], there was no significant difference in effort regulation between preclinical and clinical year students. Beyond that, we observed a different trend in our study, with effort scores being higher for the preclinical years compared to the clinical years. This discrepancy may be attributed to different operationalisations of learning strategies or to differences in underlying educational systems.

Regarding preferred media and tools, there was a significantly lower use of textbooks and other literature in the clinical years (12% vs. 36% in preclinical years). This is cause for concern in a way that advanced students appear to be less interested in acquiring in-depth knowledge and reading material that may not be imminently useful for exams. The strong emphasis on resources that will help students pass exams is understandable, but as this approach limits the breadth of content students are being exposed to, it may prove detrimental for clinical medicine and in fact life-long learning.

### Practical Implications for Medical Education

Some of the findings reported here may be of particular interest to our medical school but if confirmed in other samples, they hint at general challenges medical education is faced with. The following suggestions can be derived from our results:

1. Medical educational trainings for students

Based on the lower means on the scales *critical review*, *organisation*, and *time*, possible topics for medical educational trainings could be how to develop and use effective time tables, how to create helpful and well-structured content or lecture summaries, and what the benefits are in comparison to using premade summaries. In addition to that, those trainings could inform about the difference between performance and mastery goals or different forms of motivations in general. With such trainings, awareness on different learning strategies and motivation can be raised. However, these trainings will only have an effect on learning behaviour if flanked by curricular changes encouraging a deep approach to learning.

2. More open and practice-oriented assessments

The apparent focus on studying with previous exam questions could be reduced by replacing multiple-choice exams with question formats on a higher taxonomic level. Increasing the number of OSCEs may also meet students’ ambitions for a higher practical orientation of their studies. Furthermore, open formats do not require a focus on niche topics to produce new multiple-choice questions that have not been used before. For a practical implementation, Hauer et al. [29] propose specific recommendations for generating and evaluating examinations like creating exam blueprints or standardised scoring criteria. If necessary, a possible compromise between practice orientation and efficiency can be found in the key feature approach with short-answer questions or multiple-choice questions. Key feature questions are suited to efficiently assess clinical reasoning, i.e. important decisions in the process of diagnosis and therapy [30, 31].

3. Consideration of students’ study habits in curriculum development and teaching

Finally, the students’ wish for receiving lecture material like slides to prepare or repeat lectures could serve as a possibility to influence their study and learning process. To counter the risk of focusing on memorising the bullet points on the slides, information on the slides can be reduced to increase attention and critical thinking during lectures. Lecture material should also include current research topics or scientific literature to promote scientific competencies, which according to the study mentioned earlier are valued highly by students as well [24].

### Limitations and Future Directions

Strengths of this study included the high number of participants and the use of a validated questionnaire (LIST-K; [23]). Furthermore, the gender distribution in the sample is comparable to the actual gender distribution in German medical schools considering that in 2022, 64.4% of German medical students were female according to the German Federal Statistical Office [32]. In contrast to other recent studies [20], both students in the preclinical and the clinical years were included, allowing comparisons between the two groups. However, a longitudinal design would have allowed making statements about intra-individual changes and hence should be aimed for in the future. Other limitations should be considered when interpreting the results and drawing conclusions as well. For example, this study explicitly examined medical students’ study habits operationalised by their learning strategies as well as the media and tools they used. Certainly, the learning process is not limited to preparing for lectures or exams but also includes implicit learning sites during internships and practical study sections (e.g. bedside teaching, practising consultations with simulated patients), which were not specifically addressed by the questionnaire. Future studies should examine learning in a wider sense, for example by including a number of learning sites in the survey.

Moreover, an intriguing direction for future research would be to explore the relationship between study habits, students’ performance, and exam scores. Additionally, it might be interesting to repeat this study in the later future since the results of our survey might still be influenced by the shift to digital education during the covid-19 pandemic.

Another limitation of the study is the restriction to one German university, which of course cannot be seen as representative for the whole population of medical students. The study should be extended to different universities in Germany as well as beyond. Thus, an intercultural comparison of different national educational systems and a comparison of different programmes within the same educational system can be achieved.

## Conclusion

The results of this study showed a strong alignment of student learning activities to the predominant exam format of multiple-choice exams in the sense of assessment-driven learning. A change of exam formats may positively affect the study process of students. Medical students’ study habits including learning strategies and commonly used tools can and should be considered when planning, implementing, and evaluating a curriculum.

## Supplementary Information

Below is the link to the electronic supplementary material.

Supplementary file1 (DOCX 25 KB)

## Data Availability

The data that support the findings of this study are available from the corresponding author upon reasonable request.
