# Supplementary material for: Study Habits in Medical Education: Examining How German Medical Students Study Using a Cross-sectional Mixed-Methods Survey
Source: Med Sci Educ. 2025 Feb 15;35(3):1441–9. doi: 10.1007/s40670-025-02324-9 (PMC12228932; doi:10.1007/s40670-025-02324-9)
Supplement: Supplementary file 1 — Supplementary file1 (DOCX 25 KB) [file 40670_2025_2324_MOESM1_ESM.docx]

**Appendix A**

**Table 2**. Variables including wording or exemplary item and answer options

| **Variable** | **number of Items** | **wording / *example item*** | **answer options** |
| --- | --- | --- | --- |
| gender | 1 | Please select your gender. | - male - female - non-binary/other - no response |
| semester | 1 | Please select your current semester. | - 1st Semester - 2nd Semester - 3rd Semester - 4th Semester - 5th Semester (1st Clinical Semester) - 6th Semester (2nd Clinical Semester) - 7th Semester (3rd Clinical Semester) - 8th Semester (4th Clinical Semester) - 9th Semester (5th Clinical Semester) - 10th Semester (6th Clinical Semester) |
| media and tools during semester (m&t_sem) | 1 | Which of the following media or tools do you predominantly use over the course of the semester (e.g. to prepare for and revise lectures)? | - Amboss (Content) - Amboss (IMPP questions) - ViaMedici - Online flash card applications (e.g. Anki) - Lecture materials (e.g. lecture slides) - Seminar materials - Past exam questions - Flashcards - Textbooks or other literature - Other media or tools |
| media and tools exam preparation (m&t_exam) | 1 | Which of the following media or tools do you predominantly use in preparation for the final exams? | - Amboss (Content) - Amboss (IMPP questions) - ViaMedici - Online flash card applications (e.g. Anki) - Lecture materials (e.g. lecture slides) - Seminar materials - Past exam questions - Flashcards - Textbooks or other literature - Other media or tools |
| exam_binary | 1 | Are you currently preparing for one of the state exams (M1 or M2)? | - Yes, I am currently preparing for the first state exam (M1).   → m&t_M1   - Yes, I am currently preparing for the first state exam (M2).   → m&t_M2   - No, I am currently not preparing for any of the state exams (M1 or M2)   → m&t_plan |
| m&t_M1 | 1 | Which of the following media or tools do you predominantly use in preparation for the first state exam (M1)? | - Amboss (Content) - Amboss (IMPP questions) - ViaMedici - Online flash card applications (e.g. Anki) - Lecture materials (e.g. lecture slides) - Seminar materials - Flashcards - Textbooks or other literature - Other media or tools |
| MH_Ex | 1 | Which of the following media or tools do you predominantly use in preparation for the second state exam (M2)? | - Amboss (Content) - Amboss (IMPP questions) - ViaMedici - Online flash card applications (e.g. Anki) - Lecture materials (e.g. lecture slides) - Seminar materials - Flashcards - Textbooks or other literature - Other media or tools |
| m&t_plan | 1 | Which of the following media or tools do you plan to use in preparation for the state exams? | - Amboss (Content) - Amboss (IMPP questions) - ViaMedici - Online flash card applications (e.g. Anki) - Lecture materials (e.g. lecture slides) - Seminar materials - Flashcards - Textbooks or other literature - Other media or tools |
| Study strategies | 39 | *e.g. My workspace is organized in such a way that I can quickly find everything. (LU3)* | 5-step scale from ‘very rarely’ to ‘very often’ |
